# Some Considerations Pertaining to Immediate Root Filling

**Published:** 1904-01

**Authors:** 


### SOME CONSIDERATIONS PERTAINING TO IMMEDIATE ROOT FILLING.

Read beore the New Jersey State Dental Society, July, 1903.

Immediate root filling I understand to mean the placing of a permanent root filling immediately after extirpation of a pulp prepared for removal by rasenic, pressure anæsthesia, cataphoresis or any analogous method, or immediately after opening up a root canal containing a gangrenous pulp, or remains of one, for the purpose of filling it. When a canal containing either sort of pulp is tentatively dressed with cotton after the extirpation, a later permanent filling cannot be considered as "immediate," though if tentative dressing be unavailing and canal or apical sepsis recur and permanent fillng then effect a cure, I should regard the latter procedure as an entirely new departure from the method previously employed and legitimately to be regarded as "immediate filling."

Again the opening and sterilization of a root canal for purposes of abortion in cases of acute apical abscess does not bar a subsequent sterilization and filling from being called "immediate." It is quite possible to adopt a form of removable immediate permanent root filling which may be placed in prepared root canals with the intention of leaving it in place if no pathological condition supervene, while yet it may be removed for further treatment if such condition arise. The placing of a permanent cavity filling or of a temporary one immediately after the root filling is placed has nothing whatever to do with the operation known as immediate root filling, although the first procedure may of course indicate greater confidence in the method employed.

Immediate root filling as thus defined is not a new thing, but as it is in many cases a good procedure and of vital interest to us all, I take up its consideration before you. At the October, 1902, meeting of the Philadelphia Academy of Stomatology, Dr. James G. Palmer of New York pleaded for conservatism in root canal treatment and favored a tentative root treatment particularly in septic cases and was supported by Dr. Louis Jack and others. Dr. F. Milton Smith of New York, however, took strongly the opposite ground and claimed that thorough sterilization of septic root canals and apical tissues allowed him to fill permanently all septic root canals at the first sitting. He excepted one condition, that of a canal from which he could not exclude moisture which would persistently enter by way of the apical foramen.

My early education in root canal treatment together with perhaps a certain degree of over respect for my patients inclined me to the use of the tentative method, but after hearing the discussion referred to I resolved to conduct experiments of a clinical nature to determine for myself some of the points in connection with these cases.

In the college clinic I gave instructions to students to fill roots permanently and immediately after extirpation of the pulp by cocaine pressure anæsthesia and after the use of arsenic, which method was then most commonly in use. There was almost absolute success when directions were followed and such teeth as gave discomfort were found to have done so as the result of imperfect manipulation of the root filling. In a few cases the roots were found only partly filled and proper filling produced the desired good results. For the most part in these cases cones rolled from temporary stopping were packed in sections into the canal which was moistened with eucalyptol.

The same results have been found in my own patients. In this class of cases I have used immediate filling for many years except where much apical irritation has resulted from broaching. I now, however, do not regard this as even a bar if formapercha be used, of which I shall speak further on. In cases of aborted apical abscess, the teeth were left open after venting and root canal sterilization until such time as subsidence of the apical inflammation occurred.

By this time the canals were again infected and resterilization had to be resorted to. In some of these cases the tentative method was used nd in others immediate filling was ordered employed as for uncomplicated gangrenous pulp.

In the cases of uncomplicated gangrenous pulp, the teeth were sometimes treated with the rubber dam in place and sometimes without it, as for example in cases in which the dam could not be successfully employed to exclude saliva. In the latter cases the mouth was sterilized with a strong potassium permanganate solution. Next the teeth were opened with burs until access to canals was obtained. The cavity was flushed out, the patient requested to open the mouth and the excess of moisture removed from the bulb of the pulp cavity. Previously a little sodium dioxide in powdered form was placed on a glass mixing slab and a drop of water was also placed on the slab near it. A fine Donaldson cleanser was nowd rawn though the water then through the sodium dioxide and the adherent mass carried into the root canal and gently worked in for a short distance. The sodium dioxide reacted with the water and produced sodium hydrate and hydrogen dioxide which liberated nascent oxygen. When a portion of the canal was sterilized, a Moffet syringe was held in the left hand and while gently broaching with the right hand a stream o water was passed in. The combined action freed the canal of remnants of pulp, etc., as far as the broach was inserted. The mouth was now again evacuated, the cavity again dried and more sodium introduced and carried further in. If during the course of the sterilization saliva began to enter the cavity, it was removed by a douching from the syringe and work again resumed. No attempt was made to prevent a deep action of the sodium dioxide, in fact it was invited for the fine roots, yet not carried too far into the open foramen. If possible, the foramen was explored with the broach. This work was continued for possibly ten minutes with a fairly open root; the other cases required a longer time, and, if necessary, drills and sulphuric acid were used to enlarge the root canals.

The mechanical work and sterilization completed, the mouth was napkined to exclude moisture; the roots were dried with cotton twist on tempered Swiss broaches, thoroughly dried with hot air and filled permanently, or if desired, tentatively dressed. The permanent fillings experimented with were chloropercha on cotton twists, guttap ercha cones in sections, the canal being moistened with eucalyptol, temporary stooping cones used in the same manner and in certain doubtful cases forma-percha on cotton.

In careful hands, good results were obtained and in my own practice I have enjoyed the satisfaction of having no particular trouble with the method, any resulting irritation usually passing away promptly and only occasionally iodine counter-irritation being required.

For about a year I have had the pleasure in the use of forma-percha in the canals of molars. This material was introduced by Dr. John C. Blair of Louisville, Ky., and he now states that it is composed of red base-plate gutta percha, oil of eucalyptus, oil of cassia and paraform. As it comes for use it is in a stable, thick solution or semi-solid of friable nature. It may be taken upon a spatula and gently warmed over a flame into a thick, creamy mass, which does not quickly return to the friable state. In this may be dipped a twist of cotton rolled on a fine Swiss broach, or other suitable cotton carrier, which is at once carried into the sterilized and dried canal. On the Swiss broach it is carried in with a twist to the right which screws the cotton into the canal. When placed, the broach is turned twice to the left which disengages the broach from the cotton fibres. It is withdrawn a sixteenth of an inch and the cotton crimped upon itself to pack it firmly into the root. This is repeated until the cotton is all firmly packed in. To do this successfully requires that the pointed end of the broach be cut off with scissors before rolling the cotton upon it. After this operation a cone of temporary stopping place is placed into the surplus of forma-percha remaining at the mouth of the canal. Any further surplus of forma-percha is now to be removed from the pulb of the pulp chamber by wiping out with cotton; then oxychloride of zinc, stiffly mixed, is carried in on a ball burnisher or other packer, is distributed with it and then further compressed with a pellet of bibulous paper or cotton.

As a rule I have used a temporary filling of pink base-plate gutta percha as a test filling. I have tested the removability of this root filling and find it easy of accomplishment, so that in doubtful cases it may be regarded as a therapeutic dressing, os as a permanent filling according to subsequent indication.

Human skill is not equal to the task of successfully opening all fine roots to the apical foramen, so that some such removable filling or tentative dressing must be employed in such cases. In the vast majority of even such canals, however, it will not require removal. It is not miscible with water and is therefore impervious to moisture; containing formaldehyde and essential oils, it is therapeutically antiseptic for a time at least and therefore displaces temporary antiseptics, and it is mechanically antiseptic. Whle it is not now possible to give it a twenty-year reputation, it may be said that Blair has given cotton and formapercha an eight-year one. I feel that it deserves recommendation for the troublesome class of cases I have described.

The comparison of the tentative and immediate methods in the same case deserves some notice. I will mention a few typical cases. In a lower bicuspid with gangrenous pulp I opened under rubber dam, sterilized and dressed with cotton an antiseptic. After two days the patient reported a slight soreness. I dressed again; still there was some tenderness and so on until I became disgusted. I then sterilized and filled the root permanently and applied iodine to the gum. After a slight soreness the tooth became comfortable and so remains.

An upper central incisor with a history of apical pericementitis was sterilized with sodium dioxide and dressed with cotton and an antiseptic. In a few days it was opened and a free flow of pus followed the removal of the cotton. I again sterilized for about fifteen minutes wlth sodium dioxide, forcing it well into the apical space. After drying thoroughly, I moistened the canal with eucalyptus and filled with sections of a cone of temporary stopping. Iddine was then applied to the gum. This case was absolutely comfortable from date of filling and was under observation for two months after filling. It did not form a fistula nor show any sign of pericemental irritation. In that class of cases in which effusions continually escape from the apical canal as is shown by the repeated moistening of the cotton swab, there is no doubt that from that point immediate work is of as great value as a tentative method. Deliquesced zinc chloride in very small quantity on the end of the cotton swab will as a rule promptly check the effusion, when the canal may be dried and filled. I know that this use of zinc chloride has been criticised, but I beg to report my own good results notwithstanding.

Immediate filling in cases of certain forms of apical abscess of semi-chronic or subacute nature seems good in my experience, but I am not prepared to advise its use in the violently acute cases. In cases of chronic abscess with fistula, it is good practice and at times seems to be the only method of effecting a cure though to be sure there are cases of septic cementum that are beyond a cure by this method. Aristo-paraffin, aristol and wax, and paraform and wax are other forms of removable root fillings and in the larger roots any of the forms of gutta percha are quite readily removable by means of drills; cotton and chloro-percha is the most difficult of them to remove.

The time saved by immediate root filling is enormous and in full practice it is a great comfort not to be compelled to sandwich patients into one's engaged hours for the purpose of treating teeth. The patients will come out of the proper time in spite of every precaution and the earning capacity of time is markedly lessened without a compensating remunateration from the treating. While no one would wilfully neglect the requirements of any case or treat immediately when circumstances indicate another course, this is a point of no mean importance. As stated by one of my confreres across the club sandwich, "One does a lot of little things in treating which tax his ingenuity and takes up his time and for which he does not get paid." He is a man who thinks well of himself and has a fine practice among well to do and discriminating patients so that I think my ground is fairly well taken.

